# General Population Mortality Adjustment in Survival Extrapolation of Cancer Trials: Exploring Plausibility and Implications for Cost-Effectiveness Analyses in HER2-Positive Breast Cancer in Sweden

**DOI:** 10.1177/0272989X241275969

**Published:** 2024-09-12

**Authors:** Kun Kim, Michael Sweeting, Nils Wilking, Linus Jönsson

**Affiliations:** Department of Neurobiology, Care Sciences and Society, Karolinska Institutet, Stockholm, Sweden; Health Economics, AstraZeneca Nordic AB, Stockholm, Sweden; Statistical Innovation, AstraZeneca, Cambridge, UK; Department of Oncology-Pathology, Karolinska Institute, Stockholm, Sweden; Department of Neurobiology, Care Sciences and Society, Karolinska Institutet, Stockholm, Sweden

**Keywords:** survival extrapolation, general population mortality adjustment, excess hazard models, standard parametric distribution models, economic evaluation

## Abstract

**Background:**

In economic evaluations of novel therapies, assessing lifetime effects based on trial data often necessitates survival extrapolation, with the choice of model affecting outcomes. The aim of this study was to assess accuracy and variability between alternative approaches to survival extrapolation.

**Methods:**

Data on HER2-positive breast cancer patients from the Swedish National Breast Cancer Register were used to fit standard parametric distribution (SPD) models and excess hazard (EH) models adjusting the survival projections based on general population mortality (GPM). Models were fitted using 6-y data for stage I and II, 4-y data for stage III, and 2-y data for stage IV cancer reflecting an early data cutoff while maintaining sufficient events for comparison of model estimates with actual long-term outcomes. We compared model projections of 15-y survival and restricted mean survival time (RMST) to 15-y registry data and explored the variability between models in extrapolations of long-term survival.

**Results:**

Among 11,224 patients compared with the observed registry 15-y RMST estimates across the disease stages, EH cure models provided the most accurate estimates in patients with stage I to III cancer, whereas EH models without cure most closely matched survival in patients with stage IV cancer, in which cure assumption was less plausible. The Akaike information criterion–averaged model projections varied as follows: −8.2% to +5.3% for SPD models, −4.9% to +5.2% for the EH model without a cure assumption, and −19.3% to −0.2% for the EH model with a cure assumption. EH models significantly reduced between-model variance in the predicted RMSTs over a 50-y time horizon compared with SPD models.

**Conclusions:**

EH models may be considered as alternatives to SPD models to produce more accurate and plausible survival extrapolation that accounts for general population mortality.

**Highlights:**

- Excess hazard (EH) methods have been suggested as an approach to incorporate background mortality rates in economic evaluation using survival extrapolation.
- We highlight that EH models with or without a cure assumption can produce more accurate survival projections and significantly reduce between-model variability in comparison with standard parametric distribution models across cancer stages.
- EH models may be a preferred modeling method to reduce model uncertainty in health economic modeling since models that would otherwise have produced implausible extrapolations are constrained by the EH framework.
- Reduced uncertainty in economic evaluations will enhance the application of evidence-based health care decision making.

## Introduction

Health economic evaluation is crucial for informed decision making around the implementation of new health technologies, especially in areas of rapid innovation, such as in oncological pharmacotherapy. Cost-effectiveness of a novel therapy is assessed to optimize resource allocation and promote transparency in pricing and reimbursement in health care policy.^1,2^ A cost-effectiveness analysis often relies on randomized controlled trials (RCTs) as the primary data source, but assessing lifetime effects of the novel therapy is challenging due to a limited follow-up duration. Immature data from RCTs necessitate survival extrapolation beyond the trial period, while the choice of extrapolation methods mostly affects the outcomes. In recent years, there has been a growing discussion with regard to uncertainty associated with different methodologies used for survival extrapolation in cost-effective analyses of oncology medicines, including in breast cancer.^3–7^ Population cancer registries provide a rich source of long-term mortality follow-up, which can be used to help investigate the accuracy of extrapolations.

The National Institute for Health and Care Excellence (NICE) recently updated the recommendation to incorporate alternative modeling methods that are aimed at reducing uncertainty in survival extrapolation.^8^ The standard parametric distribution (SPD) models may induce inconsistent results when the distributions are derived solely from the trial data. During the trial period, the disease-related risk predominantly drives mortality in the study population, whereas mortality from other causes (driven by age-related risk factors) becomes more prominent during the extrapolation period. As the extrapolation is projected further, the non–disease-related risk is expected to rise and eventually account for a substantial proportion or even the entirety of the all-cause mortality rate in the study population. When the extrapolation driven solely from the trial data indicates a lower risk of mortality to the study population than that of a matched general population, the projection is deemed implausible considering the long-term follow-up of oncology patients in clinical practice.

General population mortality (GPM) adjustment ensures that all-cause mortality risk in the study population is at least equal to, if not greater than, the expected mortality risk in the general population. This method is commonly referred to as excess hazard modeling, also known as the relative survival framework,^8,9^ and its ability to provide more reliable long-term all-cause survival estimates has been recently demonstrated.^10,11^ Postestimation truncation of hazard rates to prevent SPD models from falling below background mortality is an alternative approach that is sometimes applied by researchers once models have been fitted.^12^ This approach often relies on background rates based on the average age of the population in an ad hoc manner, while excess hazard modeling uses background mortality rates that are individually matched to trial participants by age, sex, and calendar year. Thus, excess hazard modeling is considered as a more principled and preferred method, as discussed extensively in van Oostrum et al.^13^

Excess hazard modeling is an instrumental and practical method to implement GPM adjustment that partitions overall mortality rates into the expected rates (of the general population) and the excess rates (due to the disease). The expected rates are obtained from GPM rates, which are commonly available from life tables matched to the study population. The excess rates are estimated from the model fitted to the trial data, which indicate the additional mortality risk incurred within the study population.

The excess mortality risk observed during the trial period is expected to diminish over time. Excess hazard (EH) models may project excess mortality rates that reflect this decreasing risk, but this requires trial data with sufficient maturity to enable the models to capture the decreasing excess rate. If the excess rate eventually converges with the expected rate, then this indicates the absence of cancer-driven excess risk, implying that the patients have reached a state of “statistical cure.”^9,14^ EH cure models can be fit that enforce this behavior in the long term. However, in the patient population with severe disease and a poor prognosis, cure may not be expected, resulting in the cancer-driven mortality risk consistently exceeding the GPM risk. Some types of cancer, such as lymphoma and leukemia, have longer survival and require lifelong oncology surveillance, during which an excess mortality persists compared with the general population over an extended period.^15,16^

This study focuses on survival extrapolation in HER2-positive breast cancer patients, a subgroup of interest following the introduction of targeted therapies such as trastuzumab.^17^ HER2 overexpression not only serves as a prognostic marker but has also become crucial for determining the suitability of anti-HER2 therapies,^18,19^ making it essential to study the impact of extrapolation methods. By combining clinical and pathologic markers, breast cancer can be classified into subgroups, such as cases in which HER2-positive tumors may be considered low risk in treatment with trastuzumab with or without endocrine therapy. Defining low risk in breast cancer necessitates factoring in patient-related elements such as comorbidities and age, as the risk of competing mortality may outweigh concerns about cancer recurrence from a patient’s perspective.^20^

In this study, we aim to assess accuracy and variability of survival extrapolations using SPD models and EH models, with and without assuming cure, using survival data on HER2-positive breast cancer patients from the National Breast Cancer Register of Sweden (NBCR).

## Methods

### Breast Cancer Data

The NBCR has collected data on primary invasive and in situ breast cancer since 2008. The register is known for high data quality for research with high completeness and coverage across regions and years.^21^ Considering the extensive follow-up and precise mortality data linked to the registry, this data source is highly appropriate for evaluating the predictive accuracy of modeling approaches. Data were extracted on HER2-positive breast cancers diagnosed between January 1, 2008, and December 31, 2020. All patients were followed until death or censored on March 19, 2022. Ethical permission has been granted to conduct the current study by the Swedish ethical review authority (Etikprövningsmyndigheten) in February 2023.

### Survival Models

We fitted survival models, separately by cancer stage, using SPD models and EH models with or without a cure assumption based on a range of distributions including exponential, Weibull, Gompertz, gamma, log-normal, log-logistic, and generalized gamma. The framework of these models is explained in Supplementary Table 1 with further details elsewhere.^10^ EH models were fitted using Swedish life tables for the expected GPM rates matched on age, sex, and calendar year. EH cure models were fitted using a mixture-cure model, which considers the relative survival as a mixture of 2 latent subpopulations: one that is cured and never experiences mortality due to the disease and the other that is uncured with nonzero excess mortality.^22^ Over time, this model leads to excess rates in the population tending toward zero. If the probability of cure is not a function of covariates, then it can be interpreted as an overall cure fraction (the proportion of the population estimated to be eventually cured of their disease if other causes of mortality were not acting on the population). The cure fraction should be interpreted cautiously since its estimation is sensitive to the distribution choice for the uncured component of the model.^23,24^ In a case in which the probability of cure is zero, EH cure models collapse to EH no cure models.

### Analysis

Baseline patient characteristics were stratified by cancer stage and summarized using counts and percentages for categorical variables and mean and standard deviation for continuous variables. Chi-square tests and analysis of variance tests with standardized mean differences were used to compare characteristics across cancer stages. The Kaplan–Meier (KM) estimator was used to calculate survival probabilities from the date of primary diagnosis to date of death with patients administratively censored at the end of the follow-up.

Models were fitted using trimmed analysis datasets at specific data cutoff points, reflecting the common practice in economic evaluations using immature trial data. Data cutoffs were selected to ensure sufficient statistical power, based on the number of events required for survival analysis (i.e., 6 y for stage I and II cancers, 4 y for stage III, and 2 y for stage IV). Model predictions were compared with the KM estimates up to the maximum follow-up in the registry. To assess the accuracy of the predictions between the alternative approaches, we compared the 15-y restricted mean survival time (RSMT) from the registry with the individual model estimates and the Akaike information criterion (AIC)–weighted average estimate, with 95% confidence intervals (CIs) derived via bootstrapping (*N* = 100). To assess variability in long-term survival predictions due to the choice of survival model (between-model variance), the between-model variance in 50-y RMST estimates between the alternative approaches was calculated. A higher between-model variance indicates greater variability in projections. Within-model variances, which pertain to stochastic error within the predictions of a single model, were reported with 95% CI per model by stage.

## Results

**Figure 1:**
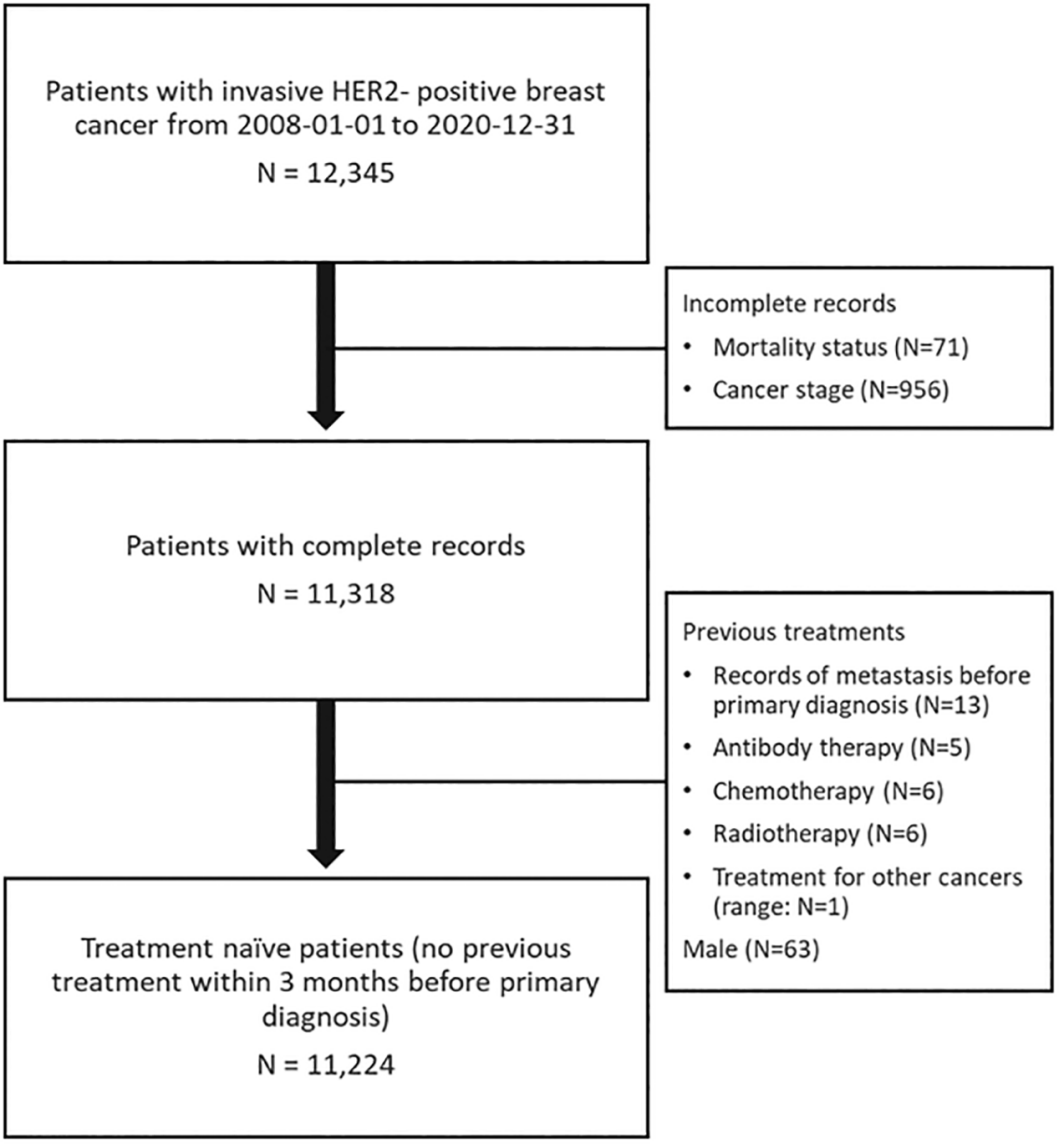
The CONSORT flow diagram.

Of 12,345 patients with invasive HER2-positive breast cancer, 1,027 patients were excluded for unknown status for mortality and cancer stage. After excluding 31 patients who received previous treatments for breast cancer or other cancers before their primary diagnosis and 63 male patients, 11,224 treatment-naïve patients were finally included. The cancer severity was defined as cancer stage I to IV, which was derived from the original records according to the TNM (tumor, node, metastasis) staging system.^25^ Cases of carcinoma in situ (stage 0) were not included in the analysis. Figure 1 illustrates the process of patient inclusion.

Table 1 presents baseline patient characteristics by cancer stage. Most of the patients were diagnosed at an early stage (stage I: 41.8%, stage II: 47.7%, stage III: 6.8%, and stage IV: 3.7%), and there was a trend of increasing age with severity of the disease, with an overall mean age of 60.2 y (*s*: 14.2 y). Most patients were postmenopausal (66.7%), estrogen receptor positive (67.4%), progesterone receptor negative (52.1%), fluorescence in situ hybridization amplified (88.4%), and had ductal carcinoma (87.7%) and Nottingham histological grade ≥2 (85.2%). Preoperation therapy including breast cancer conservation therapy, primary operation, and neoadjuvant or adjuvant antibody therapy (mostly trastuzumab) were commonly received except for stage IV patients. The median follow-up until death or censoring was 5.71 y ( $\bar{x}$ : 6.23 y) with a maximum of 14.2 y. During the follow-up period, there were 2,112 deaths (19%). Overall, there were 30.2 deaths per 1,000 person-years, and 5-y and 10-y overall survival (OS) rates were 85.1% and 74.4%, respectively, while survival rates varied significantly across cancer stages (Supplementary Figure 1).

**Table 1:** Baseline Patient Characteristics by Cancer Stage

	Overall	I	II	III	IV	*P* Test	SMD
*n* (%)	11,224 (100)	4,694 (41.8)	5,355 (47.7)	764 (6.8)	411 (3.7)		
Age, $\bar{x}$ (*s*)	60.2 (14.2)	59.6 (12.2)	60.3 (15.2)	60.7 (17.2)	63.6 (15.2)	<0.001	0.136
Menopausal status, %						<0.001	0.158
Postmenopausal	7,485 (66.7)	3,241 (69.0)	3,481 (65.0)	479 (62.7)	284 (69.1)		
Premenopausal	2,871 (25.6)	1,089 (23.2)	1,468 (27.4)	233 (30.5)	81 (19.7)		
Unknown	868 (7.7)	364 (7.8)	406 (7.6)	52 (6.8)	46 (11.2)		
Screening, %						<0.001	0.64
No	6,836 (60.9)	2,071 (44.1)	3,756 (70.1)	648 (84.8)	361 (87.8)		
Unknown	38 (0.3)	11 (0.2)	13 (0.2)	2 (0.3)	12 (2.9)		
Yes	4,350 (38.8)	2,612 (55.6)	1,586 (29.6)	114 (14.9)	38 (9.2)		
Side = right, %	5,402 (48.1)	2,287 (48.7)	2,576 (48.1)	351 (45.9)	188 (45.7)	0.38	0.037
Metastasis in sentinel lymph node, %						<0.001	1.342
No	5,594 (49.8)	3,450 (73.5)	2,078 (38.8)	39 (5.1)	27 (6.6)		
Unknown	3,453 (30.8)	360 (7.7)	2,121 (39.6)	631 (82.6)	341 (83.0)		
Yes	2,177 (19.4)	884 (18.8)	1,156 (21.6)	94 (12.3)	43 (10.5)		
Invasive site, %						<0.001	0.213
Ductal	9,847 (87.7)	4,155 (88.5)	4,705 (87.9)	652 (85.3)	335 (81.5)		
Ductal and lobular	155 (1.4)	59 (1.3)	81 (1.5)	9 (1.2)	6 (1.5)		
Ductal and other	131 (1.2)	66 (1.4)	56 (1.0)	5 (0.7)	4 (1.0)		
Lobular	572 (5.1)	251 (5.3)	262 (4.9)	39 (5.1)	20 (4.9)		
Other	408 (3.6)	152 (3.2)	197 (3.7)	34 (4.5)	25 (6.1)		
Unknown	111 (1.0)	11 (0.2)	54 (1.0)	25 (3.3)	21 (5.1)		
Nottingham histological grade, %						<0.001	0.746
1	426 (3.8)	255 (5.4)	145 (2.7)	22 (2.9)	4 (1.0)		
2	3,727 (33.2)	1,888 (40.2)	1,573 (29.4)	194 (25.4)	72 (17.5)		
3	5,834 (52.0)	2,416 (51.5)	2,951 (55.1)	355 (46.5)	112 (27.3)		
Unknown	1,237 (11.0)	135 (2.9)	686 (12.8)	193 (25.3)	223 (54.3)		
Estrogen receptor, %						<0.001	0.245
Negative	3,631 (32.4)	1,212 (25.8)	1,907 (35.6)	346 (45.3)	166 (40.4)		
Positive	7,560 (67.4)	3,477 (74.1)	3,431 (64.1)	412 (53.9)	240 (58.4)		
Unknown	33 (0.3)	5 (0.1)	17 (0.3)	6 (0.8)	5 (1.2)		
Progesterone receptor, %						<0.001	0.233
Negative	5,843 (52.1)	2,176 (46.4)	2,931 (54.7)	479 (62.7)	257 (62.5)		
Positive	5,327 (47.5)	2,504 (53.3)	2,400 (44.8)	277 (36.3)	146 (35.5)		
Unknown	54 (0.5)	14 (0.3)	24 (0.4)	8 (1.0)	8 (1.9)		
HER2 IHC test (%)						<0.001	0.652
0–1	205 (1.8)	53 (1.1)	133 (2.5)	19 (2.5)	0 (0.0)		
2	1,410 (12.6)	683 (14.6)	680 (12.7)	44 (5.8)	3 (0.7)		
3+	3,368 (30.0)	1,463 (31.2)	1,692 (31.6)	201 (26.3)	12 (2.9)		
Unknown	6,241 (55.6)	2,495 (53.2)	2,850 (53.2)	500 (65.4)	396 (96.4)		
FISH test, %						<0.001	0.201
Amplified	9,921 (88.4)	4,371 (93.1)	4,559 (85.1)	626 (81.9)	365 (88.8)		
Not amplified	246 (2.2)	83 (1.8)	139 (2.6)	17 (2.2)	7 (1.7)		
Unknown	1,057 (9.4)	240 (5.1)	657 (12.3)	121 (15.8)	39 (9.5)		
Primary treatment, %						<0.001	2.606
No operation	501 (4.5)	22 (0.5)	74 (1.4)	37 (4.8)	368 (89.5)		
Preoperative therapy	2,409 (21.5)	197 (4.2)	1,688 (31.5)	496 (64.9)	28 (6.8)		
Primary operation	8,314 (74.1)	4,475 (95.3)	3,593 (67.1)	231 (30.2)	15 (3.6)		
Antibody therapy before surgery, %	1,984 (17.7)	166 ( 3.5)	1,415 (26.4)	397 (52.0)	6 ( 1.5)	<0.001	0.800
Antibody therapy before surgery using trastuzumab, %	1,915 (17.1)	160 (3.4)	1,365 (25.5)	384 (50.3)	6 (1.5)	<0.001	0.776
Antibody therapy after surgery, %	7,371 (65.7)	3,199 (68.2)	3,670 (68.5)	497 (65.1)	5 (1.2)	<0.001	0.994
Antibody therapy after surgery using trastuzumab, %	7,251 (64.6)	3,186 (67.9)	3,583 (66.9)	477 (62.4)	5 (1.2)	<0.001	0.977

FISH, fluorescence in situ hybridization; IHC, immunohistochemistry; SMD, standard mean difference.

### Survival Analysis Using SPD Models

**Figure 2:**
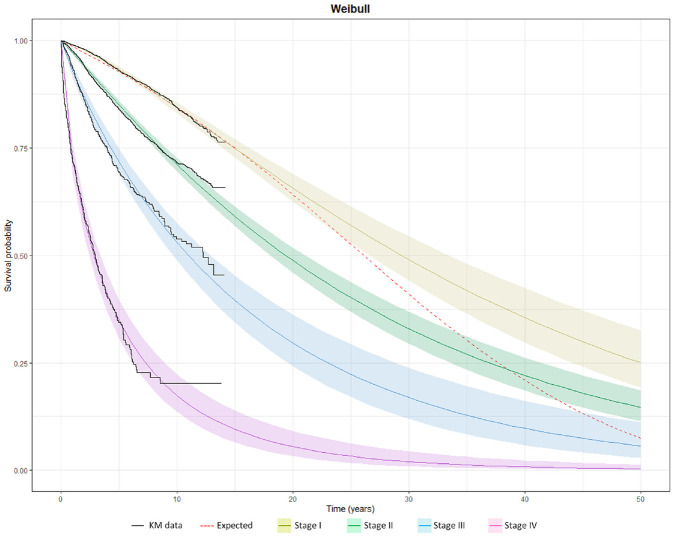
Survival by cancer stage using a standard parametric distribution (Weibull) projected over a 50-y time horizon, compared to the expected survival of the age-, gender-, and calendar year–matched Swedish life table.

To illustrate the implications of extrapolation without external information, survival data were fitted separately by cancer stage using SPD models and projected over a 50-y time horizon along with the expected survival of the matched general population across cancer stages. Figure 2 displays the survival projections using the Weibull distribution. Notably, the long-term survival projections of the early-stage cancer patients surpassed those of the matched general population, highlighting the issue with extrapolations based solely on short-term data. The Weibull models predicted a mortality rate (hazard) that plateaus for each cancer stage and drops below the GPM rate at 10, 20, 30, and 40 y for cancer stages I, II, III, and IV, respectively. Other distributions also suffered from a similar issue, with predicted long-term hazard rates that were lower than GPM rates (Supplementary Figures 2 and 3). AIC goodness-of-fit tests on the full suite of SPD models showed that the exponential and Gompertz models gave relatively poor fits (Supplementary Table 2).

### Comparison between SPD Models and EH Models with or without a Cure Assumption

**Figure 3:**
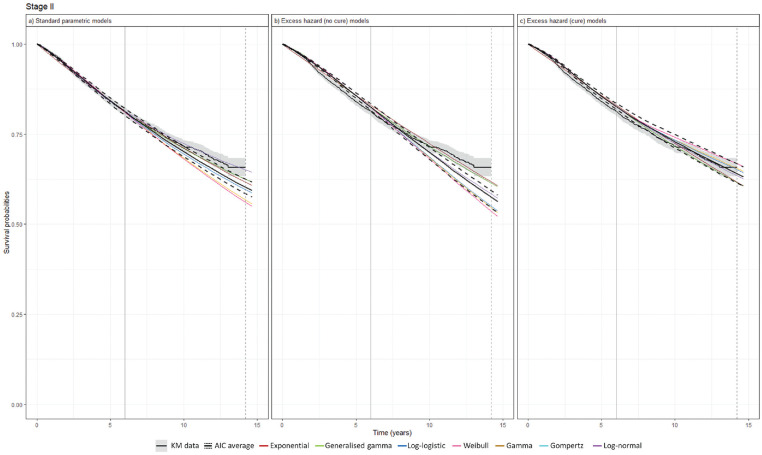
Survival extrapolation over a 15-y time horizon using standard parametric distribution models versus excess hazard models based on data cutoff at 6 y in stage II. (a) Standard parametric models. (b) Excess hazard (no-cure) models. (c) Excess hazard (cure) models. Gompertz was removed because of poor converge in the excess hazard (no-cure) model. The vertical dashed lines represent the maximum follow-up of KM data, while the vertical solid lines represent data cutoff. AIC, Akaike information criterion; KM, Kaplan–Meier.

Survival extrapolations based on immature data with early data cutoff compared with the mid-term KM data are presented for stage II in Figure 3 and for other cancer stages in Supplementary Figures 4 to 6. For the early-stage cancers with large sample sizes, these models generally aligned well with the KM data. For the late-stage cancers with smaller sample sizes, significant deviations were projected between the models. In stage II, both SPD and EH no-cure models underestimated survival by year 15, whereas EH cure models provided a closer match to the mid-term KM data. In stage I, the survival projection was similar across the models, yet EH cure models yielded the most precise reflections of the KM data. A similar pattern was observed in stage III, in which the AIC-averaged projections from SPD models and EH no-cure models underestimated 15-y survival, whereas the AIC-averaged EH cure models provided more acute predictions. In stage IV, in which the cure assumption is less plausible, the AIC-averaged EH no-cure models delivered the mid-term projections, most closely aligning with the KM data.

**Figure 4:**
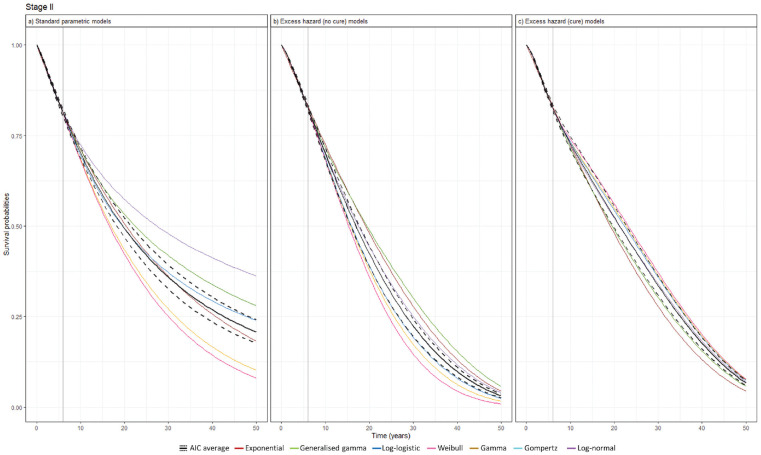
Survival extrapolation over a 50-y time horizon using standard parametric distribution models versus excess hazard models based on data cutoff at 6 y in stage II. (a) Standard parametric models. (b) Excess hazard (no-cure) models. (c) Excess hazard (cure) models. Gompertz was removed because of poor converge in the excess hazard (no-cure) model. The vertical solid lines represent data cutoff. AIC, Akaike information criterion.

Based on these models, long-term survival was projected across the cancer stages. The variance in the model projections was pronounced where SPD models projected large deviations toward the end of the projection across all cancer stages while EH models projected survival probabilities converging to zero. In stage II, although SPD models displayed small between-model variance up to midterm survival, the deviation continued to increase, resulting in large deviations by the end of the projection. Meanwhile, EH models effectively reduced between-model variance (Figure 4). This pattern was similarly observed for other cancer stages (Supplementary Figures 7–9).

Table 2 presents the RMST for each model across cancer stages, all of which were fitted to the maximum follow-up KM data from the registry. A significant reduction in between-model variance in RMST was observed when comparing SPD models to EH models with and without a cure assumption. Overall, EH models, regardless of assuming cure, tended to predict lower RMST compared with SPD models. Notably, in early-stage cancer, SPD models estimated higher RMST, such as 32.5 y for stage I, which exceeded the expected RMST of 26.0 y in the general population.

**Table 2:** Restricted Mean Survival Time (95% Confidence Interval) at 50 y by Cancer Stage

	Stage I	Stage II	Stage III	Stage IV
SPD models				
Exponential	34.3 (33.3−35.3)	24.4 (23.5−25.3)	14.8 (13.3−16.4)	4.5 (4.0−5.0)
Weibull	29.7 (27.9−31.6)	23.0 (21.7−24.4)	15.7 (13.6−18.1)	5.6 (4.7−6.8)
Gompertz	NA	25.4 (22.5−28.7)	21.1 (16.8−26.5)	10 (7.1−13.9)
Gamma	30.6 (29.1−32.2)	23.1 (21.9−24.3)	15.3 (13.4−17.4)	5.2 (4.4−6.1)
Log-logistic	32.1 (30.7−33.5)	25.9 (24.8−27.0)	18.7 (16.8−20.9)	8.8 (7.4−10.4)
Log-normal	35.2 (34.0−36.4)	28.2 (27.2−29.3)	20.3 (18.2−22.6)	9.1 (7.6−10.9)
Generalized gamma	33.0 (31.3−34.8)	26.0 (24.6−27.6)	18.1 (15.9−20.7)	7.2 (5.8−9.0)
Mean (*s*)	32.5 (2.1)	25.2 (1.8)	17.7 (2.5)	7.2 (2.1)
Min–max	29.7−35.2	23.0−28.2	14.8−21.1	4.5−10.0
Between-model variance	4.42	3.36	6.37	4.62
Excess hazard models without a cure assumption				
Exponential	24.7 (24.3−25.1)	21.4 (20.9−22.0)	15.3 (14−16.7)	4.6 (4.1−5.2)
Weibull	23.5 (22.5−24.5)	20.3 (19.4−21.1)	15.1 (13.5−16.8)	5.5 (4.7−6.5)
Gompertz	NA	20.5 (18.8−22.3)	16.2 (13.6−19.4)	6.7 (5.3−8.4)
Gamma	23.6 (22.8−24.5)	20.3 (19.6−21.1)	15.0 (13.5−16.6)	5.2 (4.5−6.0)
Log-normal	23.6 (22.8−24.5)	20.7 (20.0−21.4)	15.9 (14.5−17.3)	7.0 (6.1−8.1)
Log-logistic	24.2 (23.6−24.8)	21.3 (20.7−21.9)	16.6 (15.3−18.1)	7.3 (6.3−8.5)
Generalized gamma	23.1 (22.4−23.9)	20.6 (19.9−21.3)	15.2 (13.9−16.7)	9.1 (7.5−11.2)
Mean (*s*)	23.8 (0.6)	20.7 (0.5)	15.6 (0.6)	6.5 (1.5)
Min–max	23.1−24.7	20.3−21.4	15.0−16.6	4.6−9.1
Between-model variance	0.32	0.21	0.39	2.39
Variance change compared with SPDs	−92.8 %	−93.7%	−93.9%	−48.1%
Excess hazard models with a cure assumption				
Exponential	25.1 (24.7−25.5)	21.5 (20.8−22.2)	16.6 (13.9−19.8)	7.1 (5.9−8.7)
Weibull	25.6 (25.4−25.9)	23.8 (23.5−24.2)	19.2 (17.8−20.6)	6.8 (5.8−8.0)
Gompertz	NA	23.4 (23.0−23.8)	18.2 (16.0−20.8)	6.7 (5.3−8.4)
Gamma	24.9 (23.6−26.3)	23.2 (22.7−23.7)	18.3 (16.5−20.2)	6.2 (4.6−8.2)
Log-logistic	25.5 (25.2−25.7)	23.4 (23.0−23.7)	18.7 (17.5−20.0)	9.5 (8.9−10.1)
Log-normal	24.7 (23.4−26.1)	22.9 (22.2−23.6)	16.7 (14.3−19.5)	6.8 (5.9−7.9)
Generalized gamma	24.7 (NA)	22.9 (22.0−23.8)	17.9 (15.3−20.8)	7.4 (6.3−8.8)
Mean (*s*)	25.1 (0.4)	23.0 (0.7)	17.9 (1.0)	7.2 (1.1)
Min–max	24.7−25.6	21.5−23.8	16.6−19.2	6.2−9.5
Between-model variance	0.14	0.56	0.96	1.16
Variance change compared with SPDs	−96.7 %	−83.5%	−85.0%	−75.0%

a Gompertz was removed in stage I because of poor converge in the excess hazard (no cure) model. Excess hazard models using generalized gamma estimated the excess hazard to be nearly zero, in which the predicted survival is just the expected survival and confidence intervals are not given.

b The expected 50-year RMST in the Swedish life tables matched on age, sex, and calendar year of the cancer registry was 26.0 y.

## Discussion

EH cure models yielded the most precise estimates of mid-term survival, except for stage IV, in which a cure assumption was less plausible and EH models without cure most closely aligned with the KM data. The between-model variance in long-term extrapolation was especially large among patients with early-stage cancer whose prognosis was better. This arose since extrapolations are likely to be more challenging when survival remains high at the end of follow-up, allowing for greater room for deviation in the later-phase extrapolations.

The between-model variances in survival and RMST estimates were significantly reduced across cancer stages with EH models compared with SPD models. These estimates were derived from a large population registry cohort. However, deviations in mid- and long-term survival and RMST projections might be greater if the models are fitted to studies with smaller sample sizes, such as those from clinical trials. Although the significant reduction in between-model variances does not confirm that the survival projections from EH models are more accurate than those from SPD models given the absence of KM data for comparison over a 50-y horizon, the results suggest that EH models provide more consistent long-term survival projections. It is also challenging to trust the accuracy of SPD models given that the survival and RMST estimates for early-stage cancers are higher than those for the matched general population. Our findings align with a recent study that compared survival extrapolation with and without GPM adjustment via a relative survival framework using the Swedish cancer registry. This study finding was that for predicting 40-y survival, extrapolations from a relative survival framework corresponded more closely with observed survival from the registry, although the outcome was not stratified by cancer stage.^11^

Survival extrapolation is the main source of uncertainty in an economic evaluation, and it often leads to inconclusive decisions. Among 22 of the health economic assessment of PD-(L)1 inhibitors published by the Swedish Dental and Pharmaceutical Benefits Agency, 96% were found to be either uncertain or very uncertain, and the main source of the uncertainty (59%) was survival extrapolation.^26^ Inconclusive health economic evaluations may lead to suboptimal practice of value-based pricing and delay in access to innovative oncology medicines, which are problems not only for patients and their caregivers who may benefit from the treatments but also health care providers and health care decision makers being responsible for substantial social and economic burden of cancer diseases.^27–30^ These findings potentially suggest an alternative method to reduce uncertainty of the economic evaluation and to improve the process of information-based decision making of novel medicines.

The findings from the survival analysis of the NBCR data were comparable with the trial follow-up data of HER2-positive early-stage breast cancer patients who were treated with trastuzumab.^17^ The study reported that the 12-y OS rate was 73% (hormone receptor–positive cohort: 76%, hormone receptor–negative cohort: 70%) while we estimated a 10-y OS rate of 74.3% in overall patients, 84.4% for stage I patients, and 71.5% for stage II patients. Our study extended the long-term follow-up outcomes for the late-stage patients (53.8% 10-y OS rate for stage III patients and 20.4% for stage IV patients).

Our study has the following limitations. First, due to lack of data, we did not investigate projections for relapse-free survival (RFS) in early-stage breast cancer or progression-free survival (PFS) in late-stage breast cancer, which is another important outcome measurement of novel oncology medicines. However, we may infer that RFS/PFS extrapolation can also be improved by EH models, since there have been several studies showing a correlation between OS and RFS/PFS.^31–33^ Second, we did not consider a spline-based method, which could be more flexible to simultaneously model the registry data and external data.^34^ Third, the impact on comparative outcomes of cost-effectiveness such as incremental life-years and the incremental cost-effectiveness ratio was not investigated, which could be an area for future research. This analysis was conducted using breast cancer registry data with relatively long follow-up, which enabled us to validate mid-term extrapolations from the models. However, registry data are more limited when exploring how different methods might affect estimates of relative treatment effects.

## Conclusion

Survival extrapolation with EH models may be preferred to SPD models to reduce uncertainty in economic evaluations when the study population is adequately matched with the general population. Our findings suggest that the most plausible scenarios with survival extrapolations are provided by EH models with or without a cure assumption. EH cure models may be considered for patients with a favorable prognosis, while EH models may be considered for patients with a poor prognosis.

## Supplemental Material

sj-docx-1-mdm-10.1177_0272989X241275969 – Supplemental material for General Population Mortality Adjustment in Survival Extrapolation of Cancer Trials: Exploring Plausibility and Implications for Cost-Effectiveness Analyses in HER2-Positive Breast Cancer in Sweden Supplemental material, sj-docx-1-mdm-10.1177_0272989X241275969 for General Population Mortality Adjustment in Survival Extrapolation of Cancer Trials: Exploring Plausibility and Implications for Cost-Effectiveness Analyses in HER2-Positive Breast Cancer in Sweden by Kun Kim, Michael Sweeting, Nils Wilking and Linus Jönsson in Medical Decision Making
