# Supplementary material for: General Population Mortality Adjustment in Survival Extrapolation of Cancer Trials: Exploring Plausibility and Implications for Cost-Effectiveness Analyses in HER2-Positive Breast Cancer in Sweden
Source: Med Decis Making. 2024 Sep 12;44(7):843–53. doi: 10.1177/0272989X241275969 (PMC11491040; doi:10.1177/0272989X241275969)
Supplement: sj-docx-1-mdm-10.1177_0272989X241275969 – Supplemental material for General Population Mortality Adjustment in Survival Extrapolation of Cancer Trials: Exploring Plausibility and Implications for Cost-Effectiveness Analyses in HER2-Positive Breast Cancer in Sweden [file sj-docx-1-mdm-10.1177_0272989X241275969.docx]

# **Supplemental data**


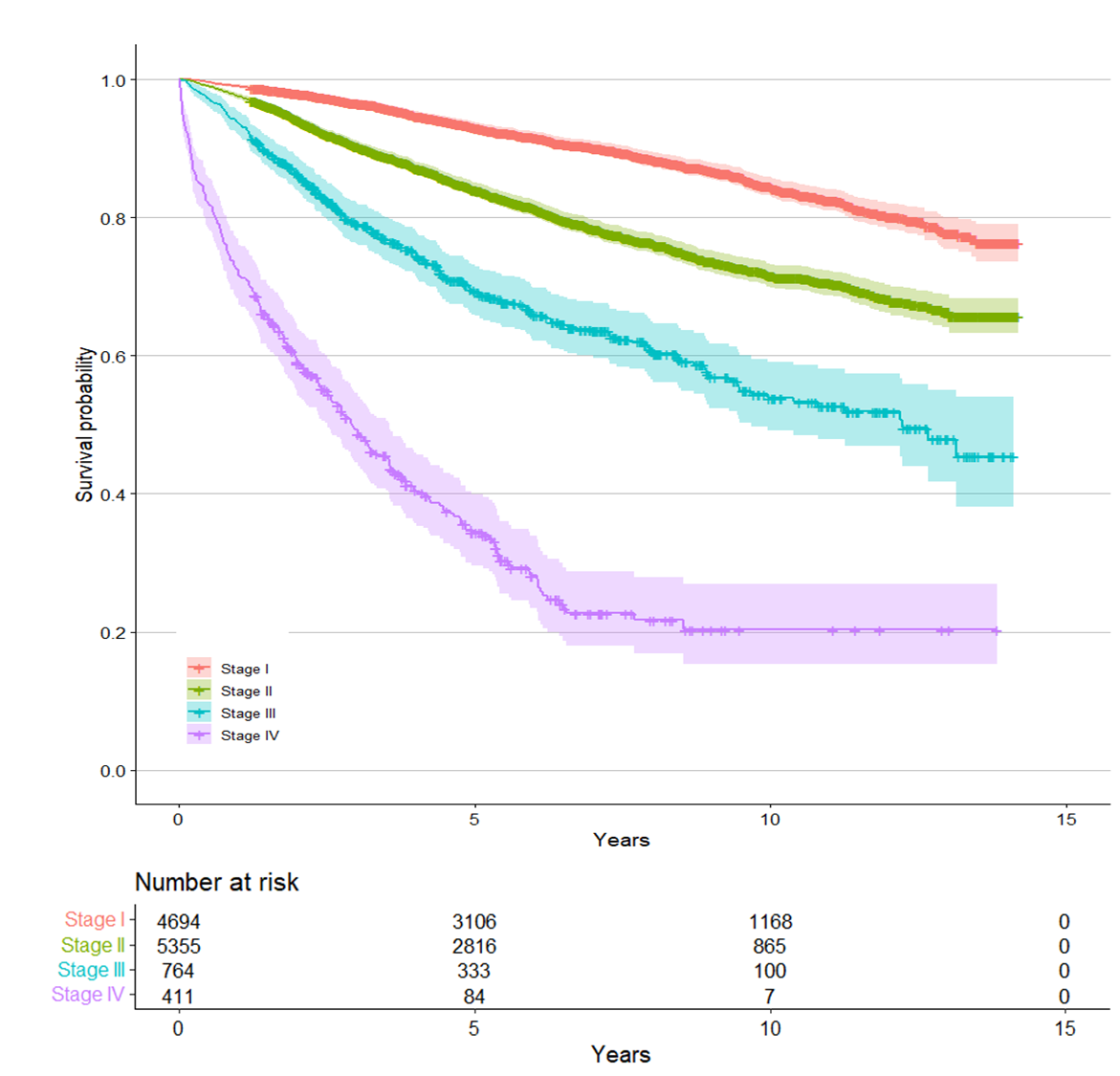


| Overall survival rate at | All | Stage I | Stage II | Stage III | Stage IV |
| --- | --- | --- | --- | --- | --- |
| 5-year | 85.1% | 93.0% | 84.0% | 69.5% | 34.4% |
| 10-year | 74.4% | 84.5% | 71.5% | 53.9% | 20.3% |

## Supplemental Figure 1. Survival in invasive HER2-positive breast cancer patients during 2008 – 2020


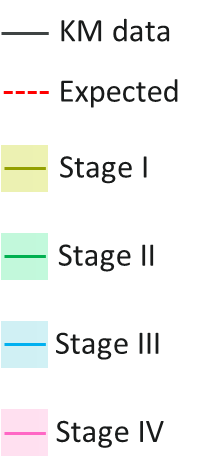

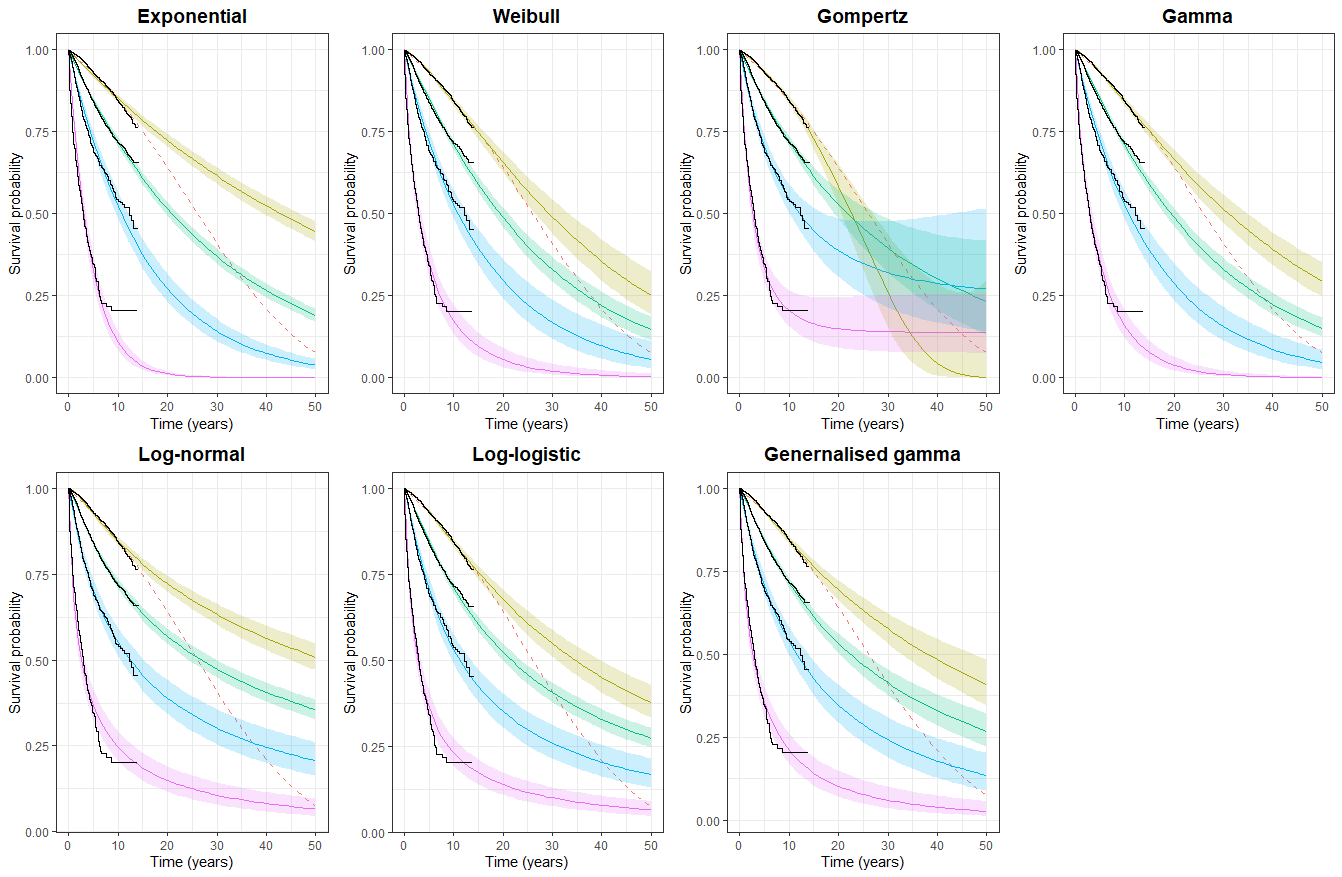


## Supplemental Figure 2. Survival plots stratified by cancer stage using a suite of standard parametric distributions projected over a 50-year time horizon


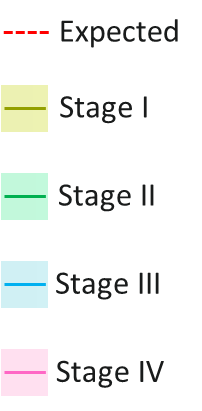

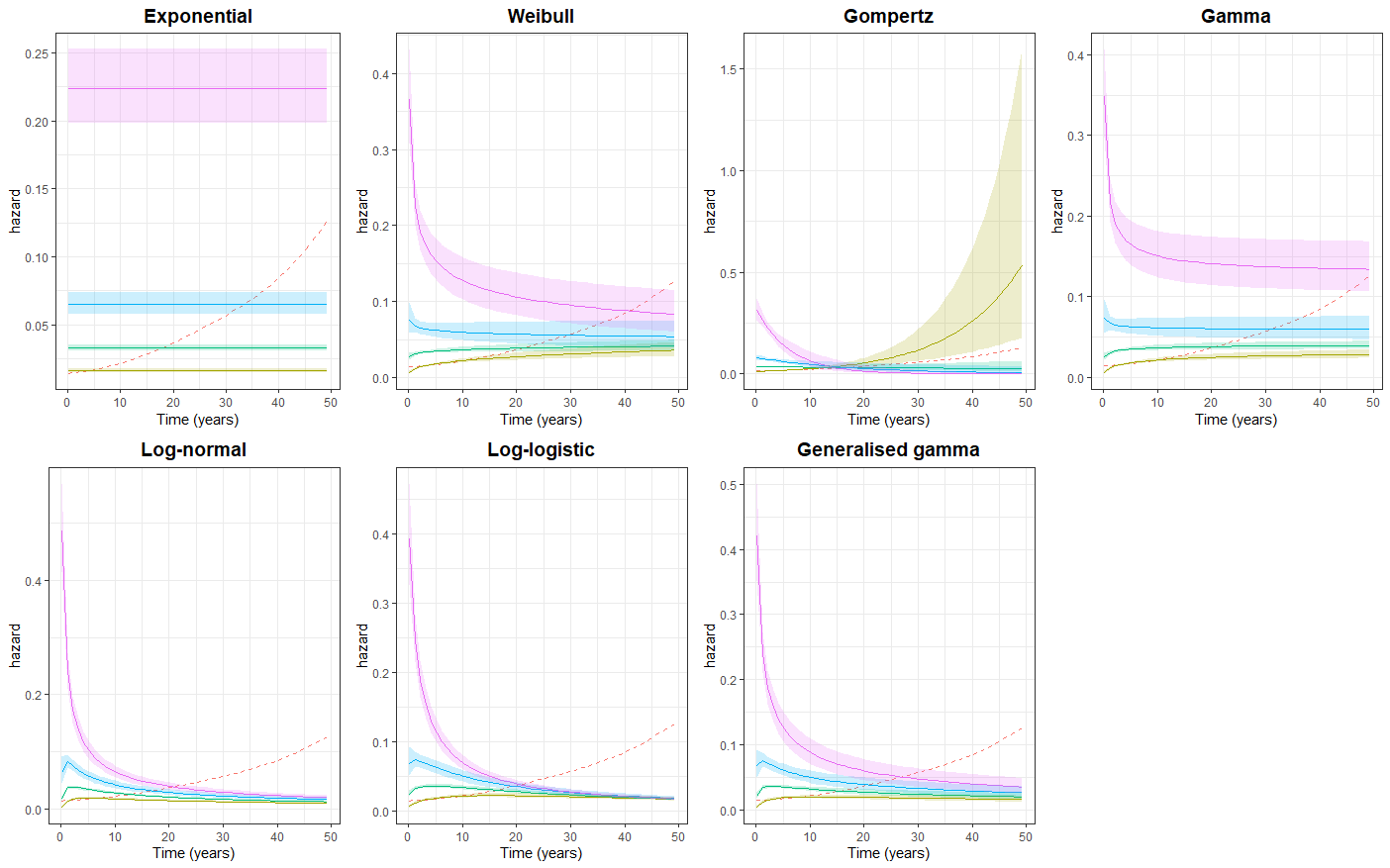


## Supplemental Figure 3. Hazard plots stratified by cancer stage using a suite of standard parametric distributions projected over a 50-year time horizon


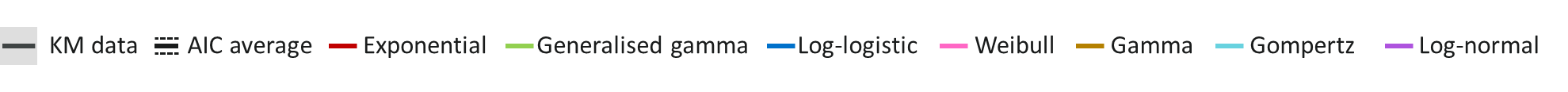

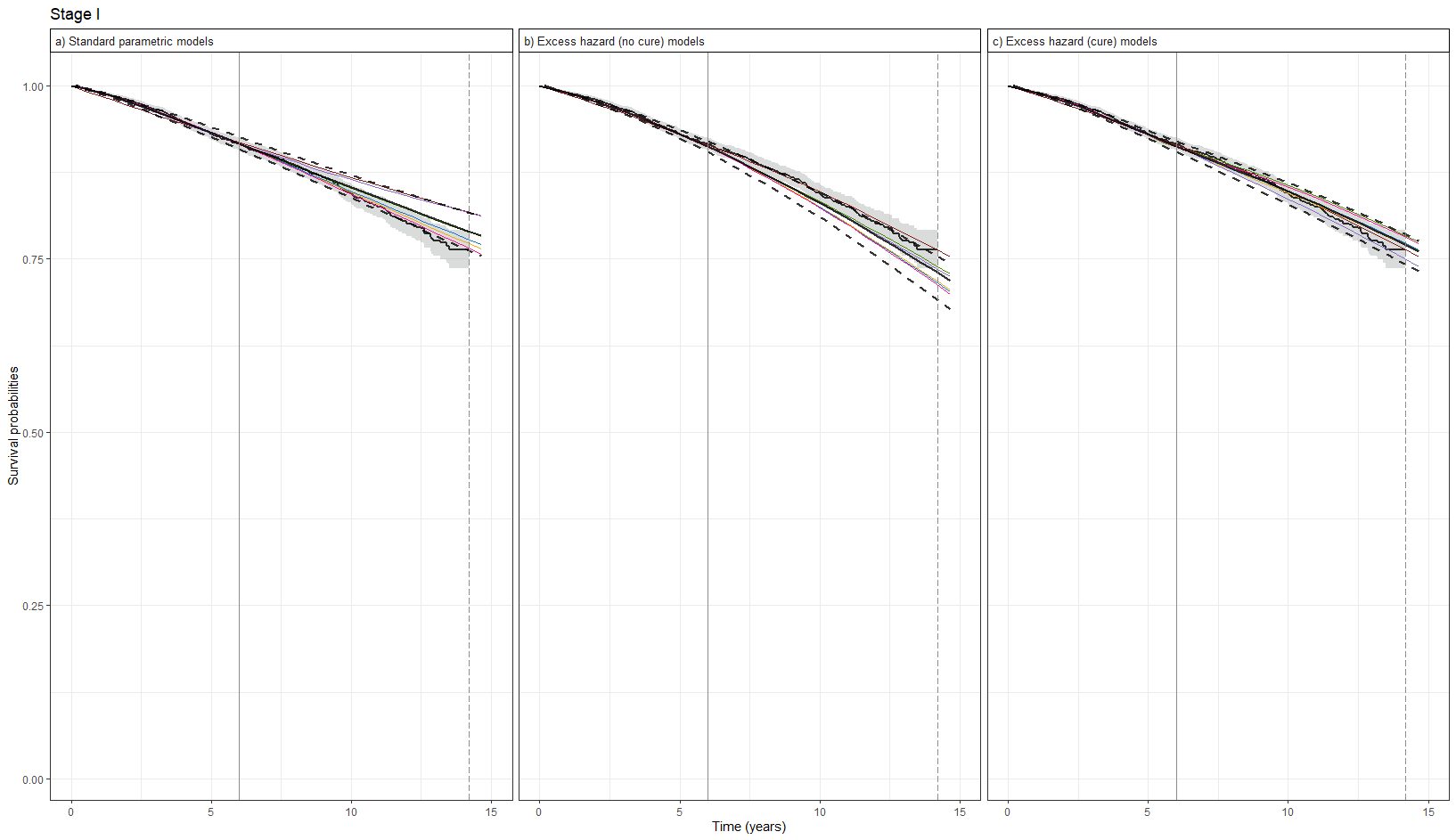


## Supplemental Figure 4. Survival extrapolation over a 15-year time horizon using standard parametric distribution models vs. excess hazard models based on data cut-off at 6 years in stage I

*Gompertz was removed because of poor converge in the excess hazard (no cure) model. The vertical dashed lines represent the maximum follow-up of KM data while the vertical solid lines represent data cut-off.


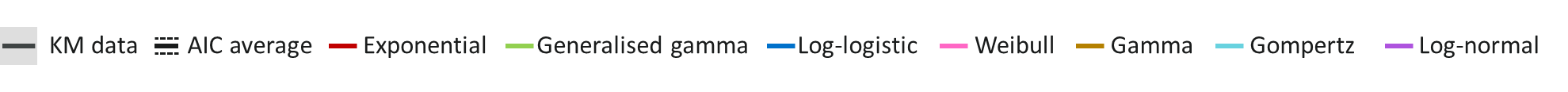

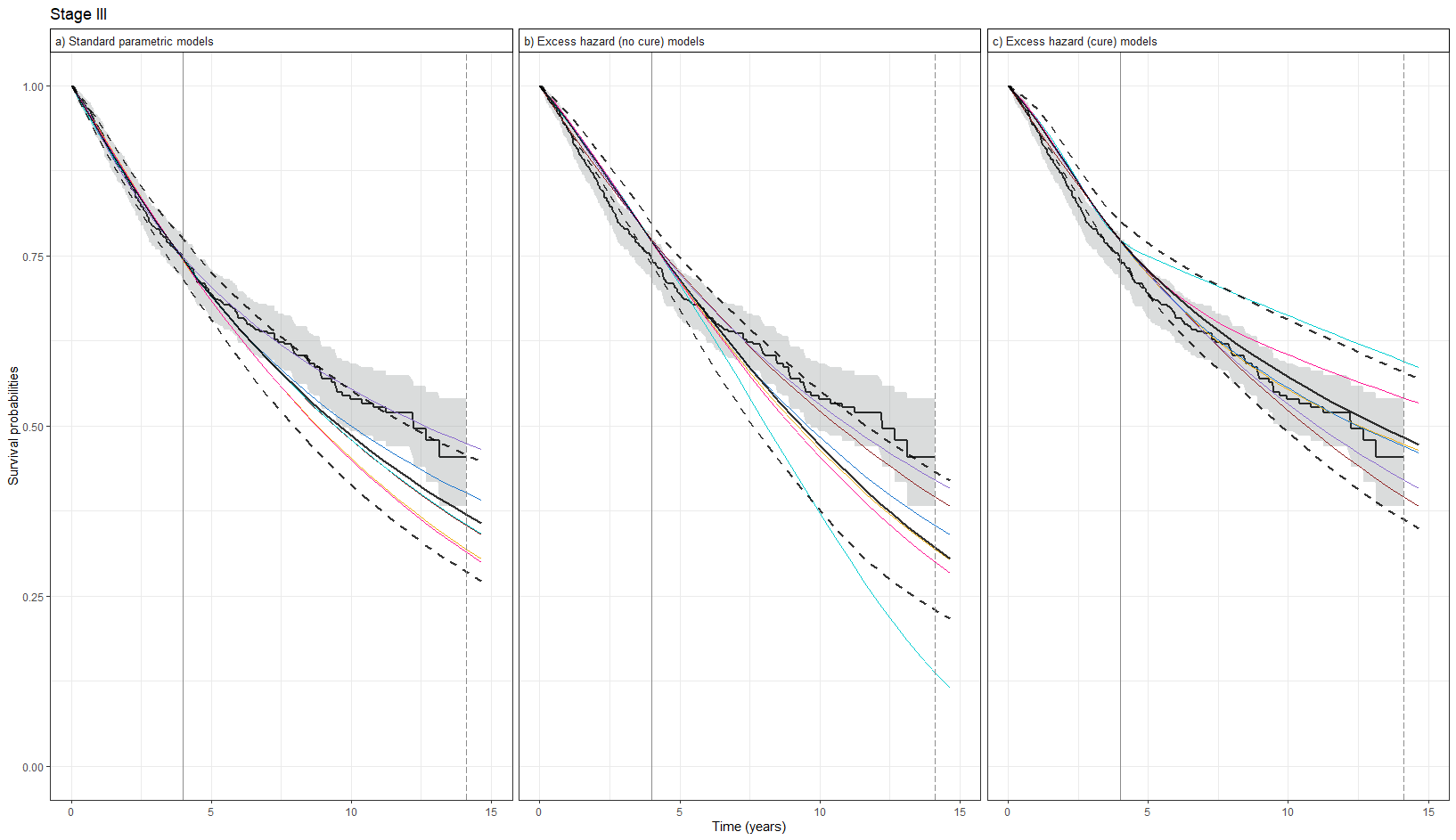


## Supplemental Figure 5. Survival extrapolation over a 15-year time horizon using standard parametric distribution models vs. excess hazard models based on data cut-off at 4 years in stage III

*Generalised gamma was removed because of poor converge in the standard parametric distribution model and excess hazard (cure) model. The vertical dashed lines represent the maximum follow-up of KM data while the vertical solid lines represent data cut-off.


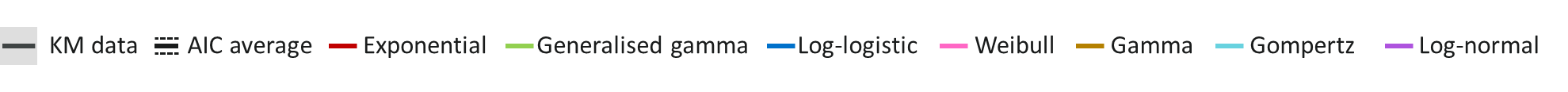

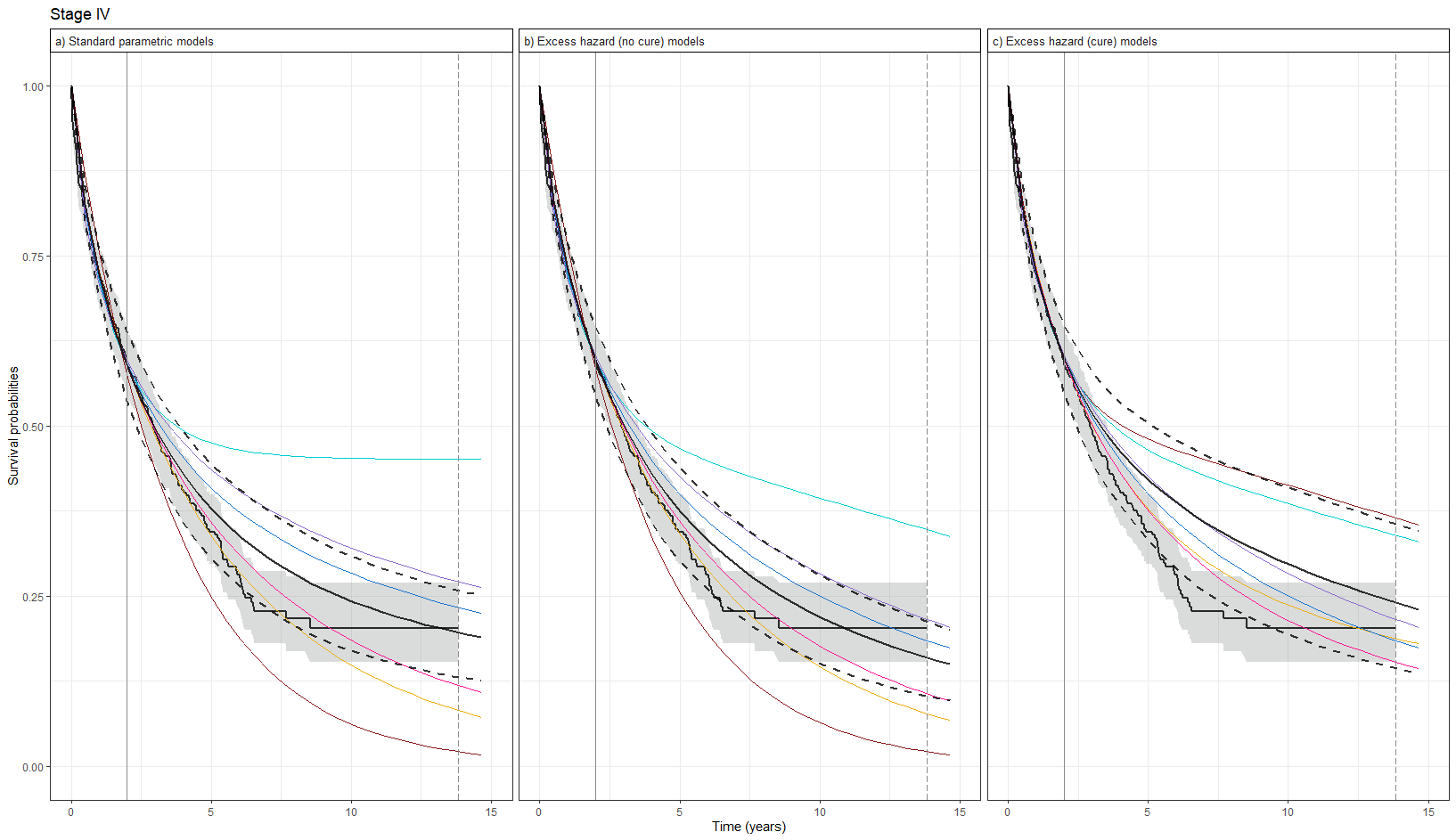


##
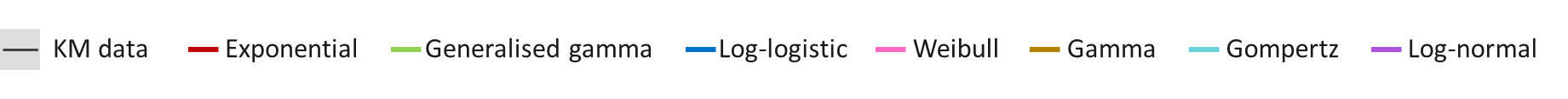
Supplemental Figure 6. Survival extrapolation over a 15-year time horizon using standard parametric distribution models vs. excess hazard models based on data cut-off at 2 years in stage IV

*Generalised gamma was removed because of poor converge in the standard parametric distribution model and excess hazard (cure) model. The vertical dashed lines represent the maximum follow-up of KM data while the vertical solid lines represent data cut-off.


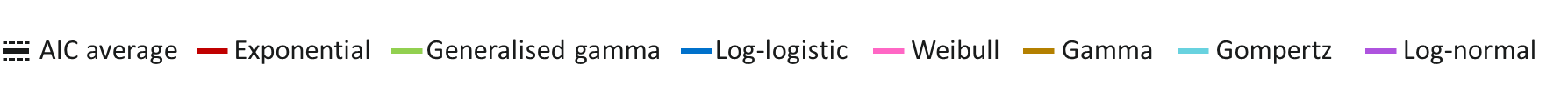

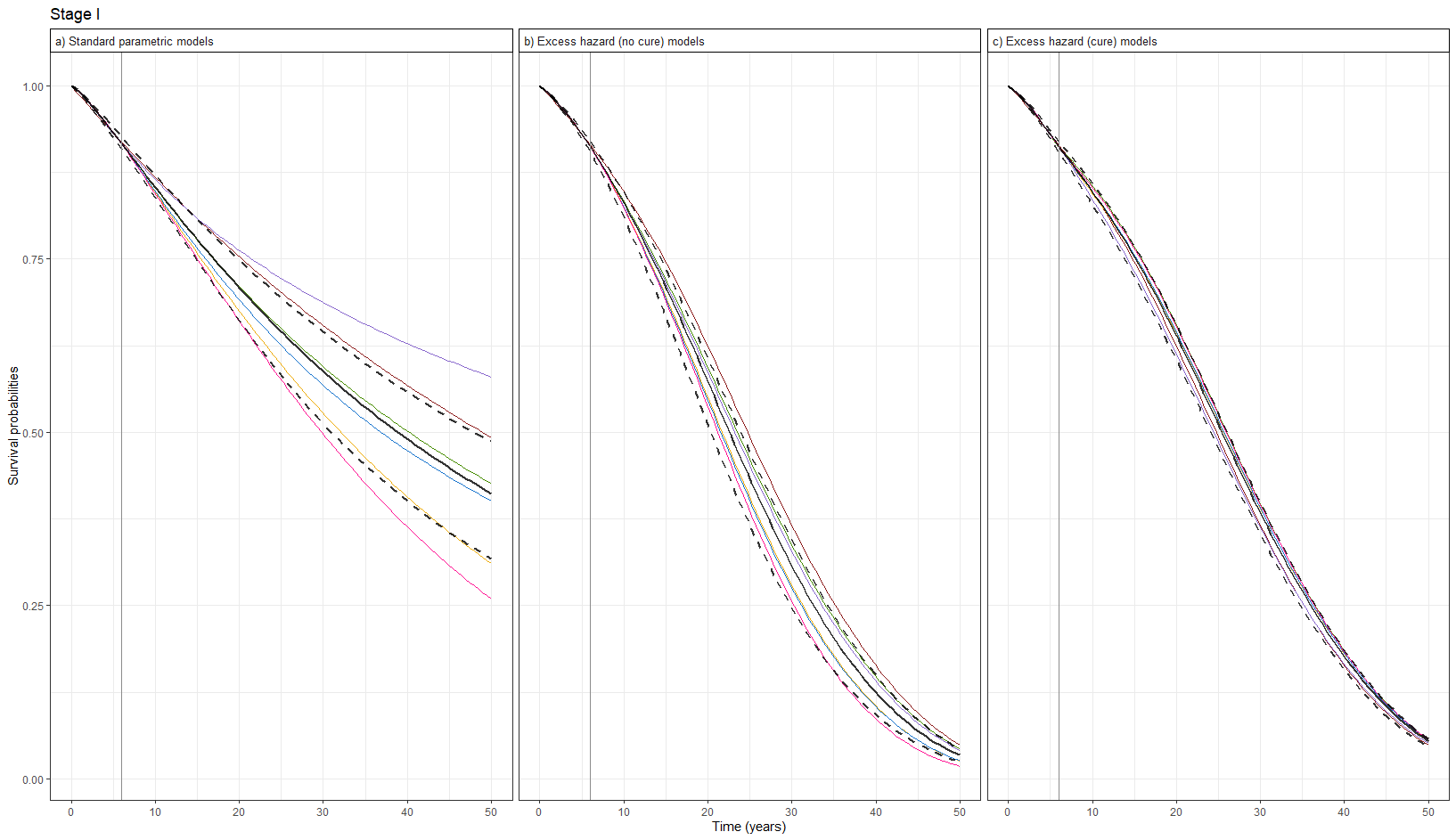


## Supplemental Figure 7. Survival extrapolation over a 50-year time horizon using standard parametric distribution models vs. excess hazard models based on data cut-off at 6 years in stage I

*Gompertz was removed because of poor converge in the excess hazard (no cure) model. The vertical solid lines represent data cut-off.


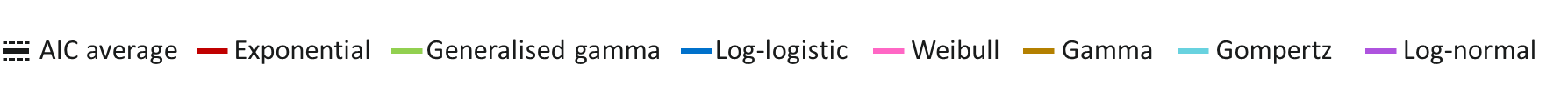

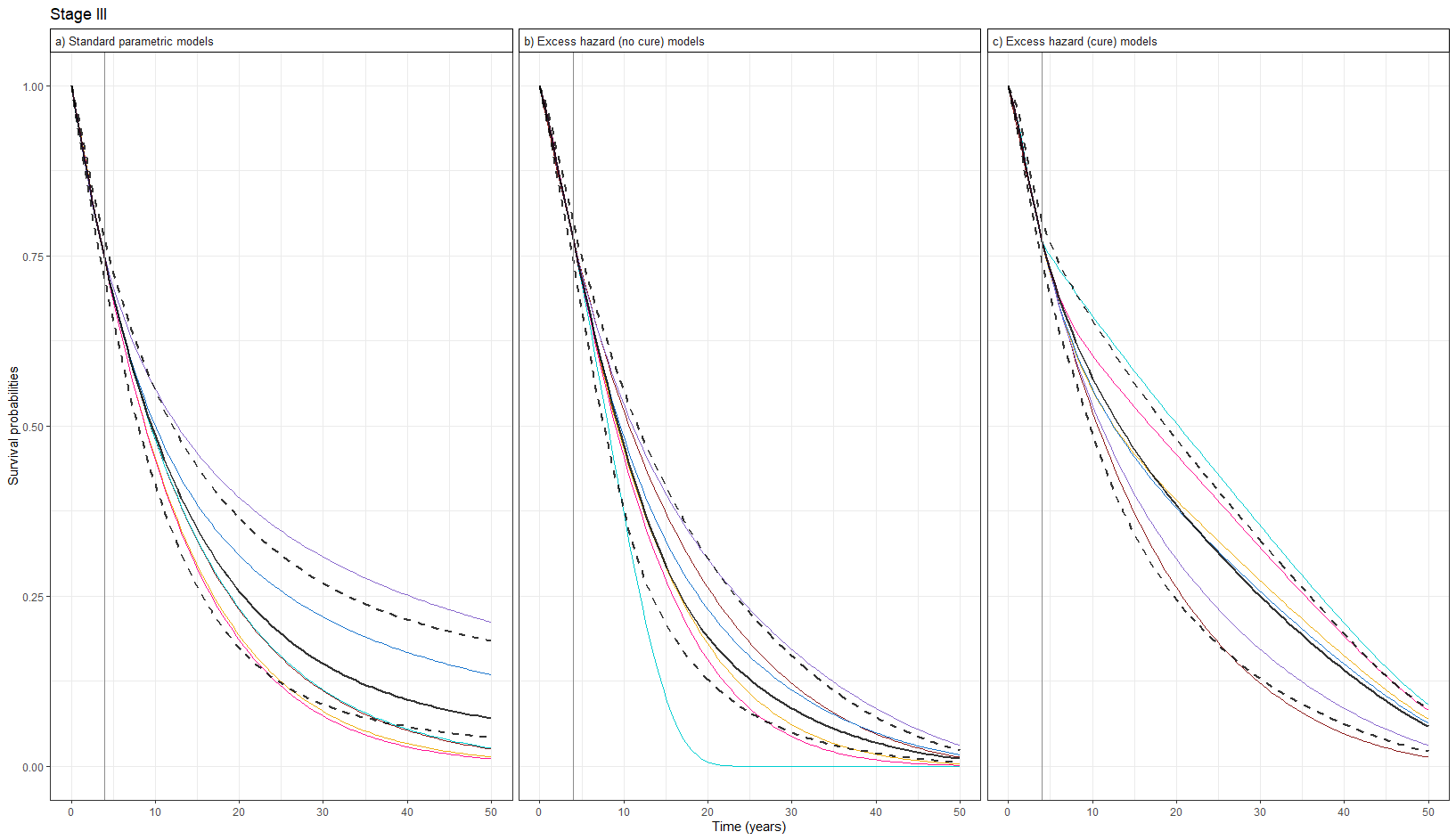


##
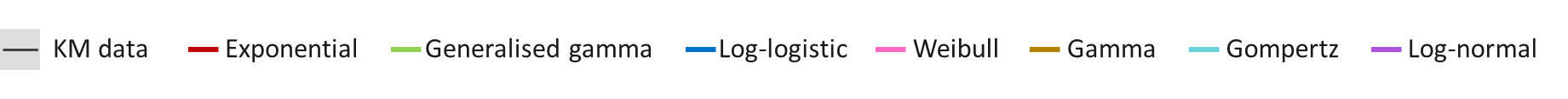
Supplemental Figure 8. Survival extrapolation over a 50-year time horizon using standard parametric distribution models vs. excess hazard models based on data cut-off at 4 years in stage III

*Generalised gamma was removed because of poor converge in the standard parametric distribution model and excess hazard (cure) model. The vertical solid lines represent data cut-off.


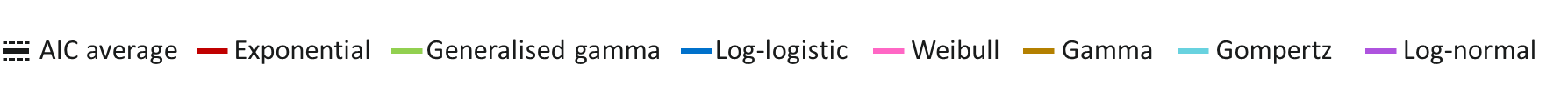

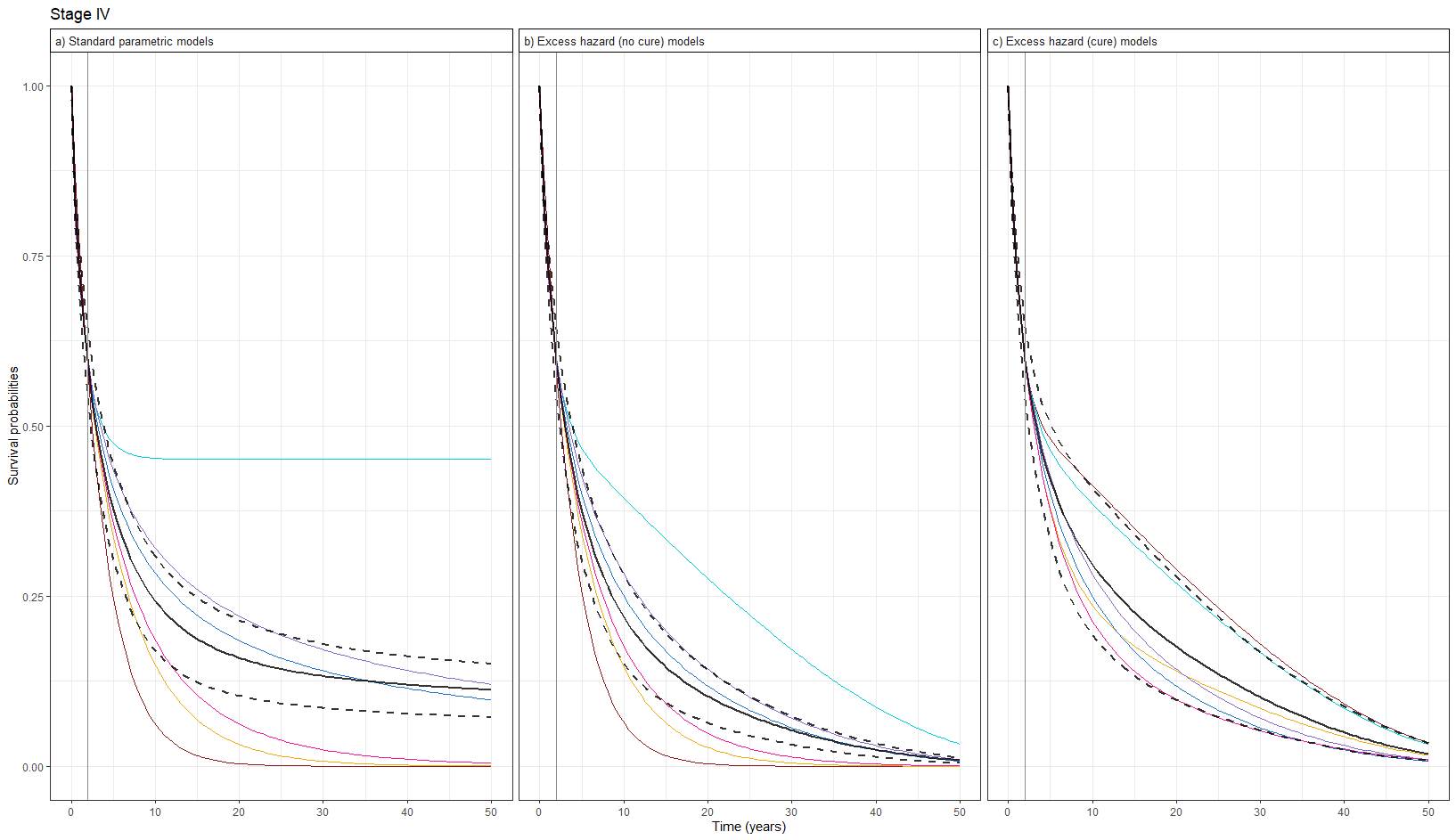


## Supplemental Figure 9. Survival extrapolation over a 50-year time horizon using standard parametric distribution models vs. excess hazard models based on data cut-off at 2 years in stage IV

*Generalised gamma was removed because of poor converge in the standard parametric distribution model and excess hazard (cure) model. The vertical solid lines represent data cut-off.

## Supplemental Table 1. Hazard and survival functions of standard parametric distribution models and excess hazard models with or without a cure assumption

| Approach | Hazard function | Survival distribution | Explanation |
| --- | --- | --- | --- |
| Standard parametric distribution models | $h\left( t \right)$ | $S\left( t \right)$ | The hazard function is directly modelled by a parametric distribution such as Exponential $h\left( t \right)=\lambda$, Weibull $h\left( t \right)=\lambda\gamma t^{\gamma-1}$, or Gompertz $h\left( t \right)=\lambda\exp(\gamma t)$. Other distributions include the Gamma, Log-logistic, Log-normal, and Generalized-Gamma distributions. |
| Excess hazard models without a cure assumption (EH no cure model) | $h_{i}\left( t \right)= h_{i}^{*}\left( t \right)+\lambda_{i}\left( t \right)$ | $S_{i}(t) = S_{i}^{*}\left( t \right)R_{i}(t)$ | The all-cause mortality rate for an individual $i$ in the study population, $h_{i}(t)$, is broken into two constituent parts, the background mortality rate $h_{i}^{*}(t)$ and the excess mortality rate $\lambda_{i}(t)$. The excess mortality rate is modelled using a SPD. Using the transformation between the hazard and survival scale, the all-cause survival $S_{i}(t)$ is a product of the expected survival $S_{i}^{*}(t)$ and the relative survival, $R_{i}(t)$ |
| Excess hazard models with a cure assumption  (EH cure model) | $h_{i}\left( t \right)=h_{i}^{*}\left( t \right)+\lambda_{i}\left( t \right)$  $=h_{i}^{*}\left( t \right)+\frac{\left( 1-\pi_{i} \right)f_{u,i}(t)}{\pi_{i}+\left( 1-\pi_{i} \right)S_{u,i}(t)}$ | $S_{i}\left( t \right)= S_{i}^{*}\left( t \right)R_{i}(t)$  $=S_{i}^{*}\left( t \right)\left( \pi_{i} + \left( 1-\pi_{i} \right)S_{u,i}\left( t \right) \right)$ | The all-cause survival probability for an individual $i$ is written as the product of the expected survival $S_{i}^{*}(t)$ and the relative survival, $R_{i}(t)$. The relative survival is expressed as a mixture-cure model where $\pi_{i}$ is the probability that the individual will be cured of their disease and $S_{u,i}(t)$ is a parametric survival function, modelled using a SPD, for the uncured component of the mixture. |

*SPD models and EH models were fitted using R packages, flexsurv and flexsurvcure. Post-estimation predictions of all-cause survival, hazard, and excess hazards were calculated using flexsurv::standsurv function.

## Supplemental Table 2. Results of Akaike information criterion of standard parametric distributions fitted to the breast cancer registry data

| Distributions | Stage I | | Stage II | | Stage III | | Stage IV | |
| --- | --- | --- | --- | --- | --- | --- | --- | --- |
|  | df | AIC | df | AIC | df | AIC | df | AIC |
| Exponential | 1 | 5496.226 | 1 | 9246.29 | 1 | 1942.092 | 1 | 1334.899 |
| Weibull | 2 | 5454.626 | 2 | 9241.104 | 2 | 1942.615 | 2 | 1296.457 |
| Gompertz | 2 | 5461.277 | 2 | 9247.887 | 2 | 1936.882 | 2 | 1310.606 |
| Gamma | 2 | 5455.177 | 2 | 9238.516 | 2 | 1943.327 | 2 | 1297.82 |
| Log-normal | 2 | 5474.137 | 2 | 9213.464 | 2 | 1933.725 | 2 | 1311.985 |
| Log-logistic | 2 | 5456.202 | 2 | 9224.116 | 2 | 1935.335 | 2 | 1304.35 |
| Generalised gamma | 3 | 5456.074 | 3 | 9212.692 | 3 | 1934.712 | 3 | 1298.329 |
